# Cloning, Characterization, and Expression Analysis of the DEAD-Box Family Genes, *Vasa* and *PL10*, in Pacific Abalone (*Haliotis discus hannai*)

**DOI:** 10.3390/genes16030329

**Published:** 2025-03-11

**Authors:** Fei Chen, Wenwei Wu, Min Li, Ying Su, Miaoqing Huang, Xuan Luo, Weiwei You, Caihuan Ke

**Affiliations:** 1 College of Biological Science and Engineering, Fuzhou University, Fuzhou 350108, China; 2 State Key Laboratory of Mariculture Breeding, College of Ocean and Earth Sciences, Xiamen University, Xiamen 361102, China; 2021401222@xmu.edu.cn (W.W.); suying1118@xmu.edu.cn (Y.S.); huangmq@xmu.edu.cn (M.H.); xluo@xmu.edu.cn (X.L.); wwyou@xmu.edu.cn (W.Y.); 3 Fujian Key Laboratory of Genetics and Breeding of Marine Organisms, College of Ocean and Earth Sciences, Xiamen University, Xiamen 361102, China; 4 Department of Otorhinolaryngology, Guangxi Hospital Division of The First Affiliated Hospital, Sun Yat-sen University, Nanning 530000, China; liminyuri@163.com

**Keywords:** DEAD-box protein family, germline, primordial germ cells, epigenesis, embryonic development

## Abstract

Background/Objectives: *Vasa* and *PL10* belong to the DEAD-box protein family, which plays crucial roles in various cellular functions, such as DNA replication, DNA repair, and RNA processing. Additionally, DEAD-box family genes have also been identified as being related to gonadal development in many species. However, the function of *vasa* and *PL10* in abalone is poorly understood on a molecular level. Methods: In the present study, we individually isolated and characterized the *vasa* and *PL10* orthologs in *Haliotis discus hannai* (*Hdh-vasa* and *Hdh-PL10*). We also characterized the mRNA distributions of *vasa* and *PL10* in various tissues from adult organisms and different embryonic developmental stages using real-time PCR (RT-qPCR) techniques. Furthermore, spatial and temporal expression of *Hdh-vasa* and *Hdh-PL10* throughout embryonic and larval development was examined by whole-mount in situ hybridization (WMISH). Results: The two predicted amino acid sequences contained all of the conserved motifs characterized by the DEAD-box family. Homology and phylogenetic analyses indicate that they belong to the vasa and PL10 subfamilies. We found that *vasa* and *PL10* mRNA were not solely restricted to gonads but were widely expressed in various tissues. WMISH showed that *Hdh-vasa* and *Hdh-PL10* largely overlapped, with both being maternally expressed and specifically localized to the micromere lineage cells during early cleavage stages. By the gastrulation stage, *Hdh-vasa* were expressed strongly in two bilaterally symmetrical paraxial clusters, but *Hdh-PL10* was dispersed in entire endodermal region. Our results suggest that *Hdh-vasa*-expressing cells are located as a subpopulation of undifferentiated multipotent cells that express *Hdh-PL10*. As such, we infer that primordial germ cells are specified from these *vasa*-expressing cells at some point during development, and inductive signals (epigenesis) play an important role in specifying primordial germ cells (PGCs) in *H. discus hannai*. Conclusions: This study provides valuable insights into the molecular characteristics and expression patterns of *Hdh-vasa* and *Hdh-PL10*, contributing to a better understanding of their roles in germ cell specification and early embryonic development in *H. discus hannai*.

## 1. Introduction

In recent years, various “germline factors” that regulate the differentiation of germ cells have been identified. Among these, the genes of the *DDX4*/*vasa* and *Ded1*/*DDX3*/*PL10* subfamilies, which are evolutionarily related, have been studied extensively [1,2,3,4,5,6]. *DDX4*/*vasa* and *Ded1*/*DDX3*/*PL10* are genes that encode DEAD-box ATP-dependent RNA helicases, which are defined by nine conserved motifs, including the D-E-A-D (Asp-Glu-Ala-Asp) sequence, from which the family derives its name. [7]. These DEAD-box genes, found in all eukaryotes and most prokaryotes [8,9,10,11,12], are involved in diverse cellular processes including splicing, rRNA processing, RNA editing, translational initiation, mRNA degradation, and nuclear mRNA export [13].

DDX4/vasa proteins, which were originally identified in *Drosophila* [14], are expressed in both male and female germ cells [15]. Studies in both model and non-model organisms have demonstrated that *vasa* plays crucial roles in germline specification and gamete development across various Metazoan species. [1,16,17]. Therefore, *vasa* has long been regarded as a key molecular marker for research on germline development. It is important to note that *vasa* is only a member of a wider regulatory network responsible for germline maintenance and specification. This regulatory network also includes another DEAD-box gene, *PL10* [18], which is important for germ cell specification in a wide range of eukaryotes, including *Hydra*, *planaria*, *Drosophila*, and mammals [19,20,21,22]. However, increasingly, studies have highlighted that *vasa* and *PL10*, previously known as germline markers, are also expressed in multipotent stem cells with the potential to generate both somatic and germline lineages [2,3,4,5,23,24,25]. Research on the expression of these “germline factors” outside of the germline has sparked a reevaluation of the traditional germline/soma paradigm [26].

Pacific abalone (*H. discus hannai*) is an economically important abalone species that is widely cultivated in China. Despite the ongoing growth in production, several critical issues, such as gonad precocity, impact the abalone farming industry [27]. To combat this problem, transient silencing of “germline factor” mRNA in mollusks has been found to impede germ cell development in the gonads. [28]. The application of these techniques requires the identification of genes associated with germ cell development, particularly those crucial for the formation of primordial germ cells (PGCs), from which germ cells arise during early organismal development [29]. DEAD-box family genes have been identified as being related to gonadal development in many mollusk species [30,31,32]. However, the expression patterns of DEAD-box family genes like *vasa* and *PL10* in abalone are still limited.

In the present study, we isolated and characterized the mRNA distribution of *vasa* and *PL10* in various tissues from adult organisms by RT-qPCR. Furthermore, we also characterized the spatial and temporal expression of *vasa* and *PL10* during embryonic and larval development using the WMISH and RT-qPCR techniques. We show here that, in *H. discus hannai*, *vasa* and *PL10* mRNA are not solely restricted to gonads but are widely expressed in various tissues, suggesting that they are not limited to animal germlines but may also participate in diverse biological processes. *Hdh-vasa* and *Hdh-PL10* exhibit significant overlap, as both are broadly expressed during early embryonic development. During late embryogenesis stages, *Hdh-vasa* and *Hdh-PL10* are restricted to specific cells. These findings imply that the primary mechanism of PGC determination in *H. discus hannai* is governed by inductive signals (epigenesis).

## 2. Materials and Methods

### 2.1. Animals and Tissue Collection

The *H. discus hannai* were grown at the Fuda Abalone Farm (Jinjiang, China). A total of six healthy adult individuals (three males and three females, one year old) were randomly collected and euthanized, and samples of several tissues (pedal, mantle, gill, hepatopancreas, blood, and intestine) were extracted and stored at −80 °C (n = 3, 3 biological replicates per tissue). The developmental phases of the ovaries and testes in healthy adult individuals (1 years old, sexually mature) were divided into four stages based on histological observation: (i) proliferation (testes/ovaries in the proliferation phase), (ii) maturation (testes/ovaries in the maturation phase), (iii) ripe (testes/ovaries in the ripe phase), and (iv) spawning (testes/ovaries in the spawning phase). Six samples from each sex were collected at each stage.

### 2.2. Embryos and Larvae Collection

Thirty reproductively mature (three-year-old) Pacific abalone (20 females and 10 males), which were randomly collected from a single site at the Fuda Abalone Farm, were induced to spawn by UV-irradiated seawater (1000 mW h/L) and temperature shock [33]. Gametes were mixed together at concentrations sufficient to allow for fertilization. According to the morphological description by Lv et al. [34], various developmental stages, identified microscopically, were sampled, as follows: unfertilized eggs; 2-cell, 4-cell, 8-cell, 16-cell, 32-cell, 64-cell, and 128-cell embryos; morulas; gastrulas; intramembranous trochophores; extramembranous trochophores; early veliger larvae; metaphase veliger larvae; and anaphase veligers. A total of 100,000 embryos or larvae were sampled for each developmental stage.

### 2.3. RNA Isolation and Reverse Transcription

Total RNA was isolated from each sample using TRIzol reagent (Thermo Fisher Scientific, Waltham, MA, USA) and treated with 0.1 Unit/µL DNase I (Sigma, Kanagawa, Japan) to remove potential DNA contamination. For reverse transcription, 1 µg of total RNA was combined with 50 ng of random hexamers and 10 mM dNTPs and then heated to 65 °C for 5 min before being rapidly cooled on ice. Reverse transcription was performed in a 25 µL reaction with SuperScript™ III Reverse Transcriptase (Invitrogen, Carlsbad, CA, USA), 5× First-Strand Buffer, 0.1 M DTT, and RNase inhibitor. The reaction was incubated at 25 °C for 10 min, followed by 50 °C for 60 min, and then heated to 70 °C for 15 min.

### 2.4. Cloning and Orthology Assignment of Vasa and PL10

Perform a BLASTX comparison of the *vasa*- and *PL10*-related cDNA sequences from various species on NCBI, prioritizing model organisms and mollusk-related sequences. Retrieve target gene sequences based on two criteria: high sequence similarity and presence of specific domains. Design primers specifically targeted the open reading frame (ORF) regions predicted by the transcriptome database. The primers for *vasa* and *PL10* were designed as shown in Table 1. The PCR products were separated by electrophoresis on a 1% (*w*/*v*) agarose gel and visualized by staining with ethidium bromide. Bands of the expected size were subsequently extracted from the gel (QIAquick Gel Extraction Kit, Qiagen, Shanghai, China), cloned with *pEASY*^®^-Blunt E1 Expression Vector (TransGene, Beijing, China), and then sequenced. Full-length ORF sequences of *vasa* (GenBank accession no: PR891392) and *PL10* (GenBank accession no: PP891393) were retrieved and analyzed using the BLAST tool (BLAST v2.11.0) in the NCBI database, confirming their orthology with other *vasa* and *PL10* genes. Multiple amino acid (aa) sequences were aligned using ClustalW. Phylogenetic analysis was performed with the neighbor-joining (NJ) method in MEGA 3.1 [35], with distance datasets subjected to 1000 bootstrap replicates.

### 2.5. Selection of Suitable Reference Genes for RT-qPCR

Before conducting an RT-qPCR analysis of target genes, it is essential to identify reference genes. The expression of reference genes should be relatively stable, as this is crucial for correcting errors that may arise during the experiment, such as variations in the initial sample amounts, cDNA synthesis efficiency, and overall transcription efficiency in cells or tissues. Reliable reference genes are crucial for obtaining accurate relative quantification data.

The selection of the reference genes for the various tissues was based on the identification of eight candidate reference genes (*ICG1*-*ICG8*) from the transcriptome data of *H. discus hannai* by calculating the coefficient of variation (CV). Two commonly used reference genes (*GAPDH* and *β-actin*) were also used as candidate genes. Primer3 [36] was used to design the specific primers according to the transcriptome database. The ten candidate reference genes were amplified from *H. discus hannai* with the primer sequences listed in Table 2. To identify the optimal reference gene(s), we systematically evaluated the expression stability of 10 reference genes in multiple tissues from adult *H. discus hannai* using RT-qPCR. An integrated assessment of gene expression stability was performed using the following three different software tools: geNorm [37], NormFinder [38], and BestKeeper [39].

The selection of the candidate reference genes for the various embryonic developmental stages was based on the identification of five candidate reference genes (*MZQ1–MZQ5*) from the transcriptome data of *H. discus hannai* by calculating the coefficient of variation (CV). Two reference genes (*UBE2* and *RPL8*) reported in a previous study [40] and three commonly used reference genes (*GAPDH*, *β-actin*, and *18S*) were also used as candidate genes. The ten reference gene candidates were isolated from *H. discus hannai* using the primer sequences provided in Table 3. To select the suitable reference gene(s) for the gene expression analysis, *H. discus hannai* embryos and larvae in different developmental stages were used as materials to detect the expression levels of ten candidate reference genes by RT-qPCR. The method for analyzing the expression stability has been described above.

### 2.6. RT-qPCR

To assess the variations in the expression levels of *Hdh-vasa* and *Hdh-PL10* quantitatively, we conducted an RT-qPCR analysis. The relative gene expression was determined using the 2^−∆∆CT^ method for quantification. According to the stability value, reference genes were selected for the various tissues and embryonic developmental stages, and these genes were further analyzed to normalize the relative expression values. RT-qPCR was performed, in triplicate, with 5 µL cDNA (1/100 dilution) in a total volume of 20 µL with each primer at 0.5 µM, 2× FastStart Universal SYBR Green Master (ROX), and nuclease-free water (4 µL). The cycling program proceeded as follows: 10 min at 95 °C, followed by 40 cycles of 10 s at 95 °C and 30 s at 59 °C. Fluorescent signal intensities were measured after the cycles were completed.

### 2.7. WMISH

For the single-labeled WMISH assays, antisense RNA probes labeled with digoxigenin (DIG) were synthesized (Roche, Mannheim, Germany). Briefly, 1 µg of DNA template was transcribed in a 20 µL reaction mixture. The reaction was maintained at 37 °C for 2 h, then treated with RNase-free DNase I to eliminate the DNA template. The probes were synthesized using the selected inserts, which were cloned into the pGEM-T vector (Promega, Madison, WI, USA). The sequences of primers for the DIG-labeled RNA probes are listed in Table 4. Fixation, storage, preparation, and WMISH for the *H. discus hannai* larvae were performed according to previous studies [41]. The DIG-labeled probes were stained with an NBT/BCIP (nitro-blue-tetrazolium/5-bromo-4-chloro-3-inodlylphosphate) substrate [42]. All specimens were captured using a Leica M165C microscope equipped with DIC optics.

### 2.8. Statistics

Statistical analyses were carried out with one-way ANOVAs in SPSS 25.0 (IBM, Tulsa, OK, USA). The significance cut-off value was considered when *p* < 0.05.

## 3. Results

### 3.1. Cloning and Characterization of H. discus hannai Vasa and PL10

**Figure 1:**
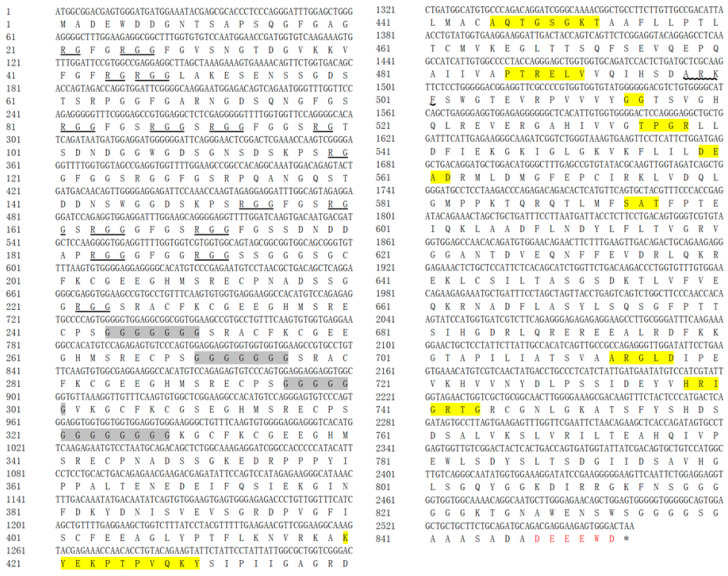
The ORF and deduced aa sequence of Hdh-vasa. Nine conserved motifs characteristic of the DEAD-box protein family (Q-motif, motif I, motif Ia, motif Ib, motif II, motif III, motif IV, motif V, and motif VI) are highlighted in yellow. RG repeats and RGG repeats are underlined. The ARKF motif is underlined with a wavy line. Four tandemly arranged glycine-enriched sequences in the N-terminal region are shaded in grey. C-terminal regions containing the conserved E, D, and W residues are indicated in red.

**Figure 2:**
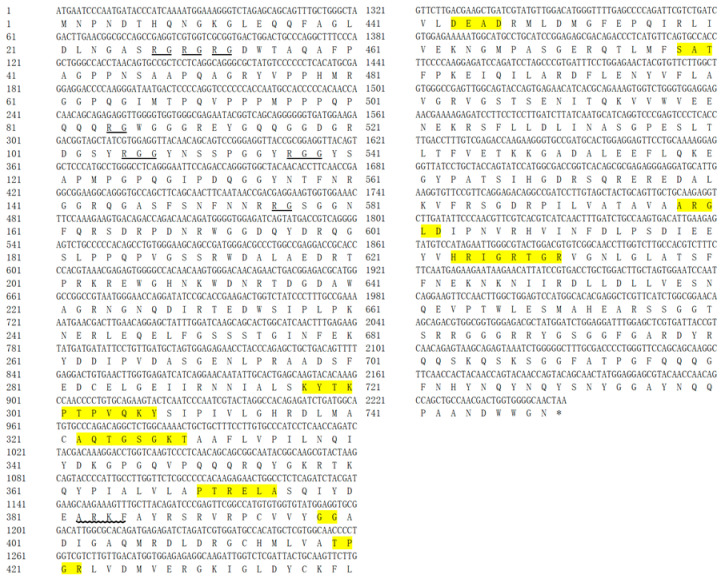
The ORF and deduced aa sequence of Hdh-PL10. Nine conserved motifs characteristic of the DEAD-box protein family (Q-motif, motif I, motif Ia, motif Ib, motif II, motif III, motif IV, motif V, and motif VI) are highlighted in yellow. RG repeats and RGG repeats are underlined. The ARKF motif is underlined with a wavy line.

**Figure 3:**
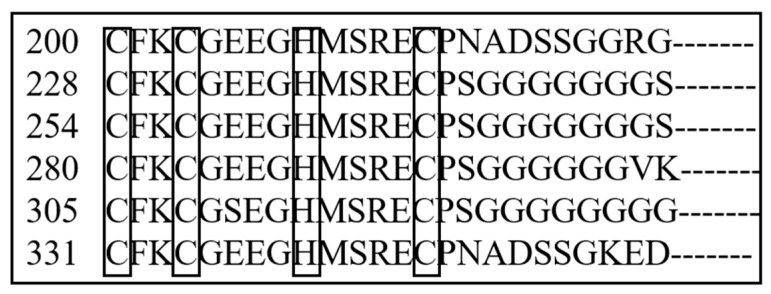
Zinc-finger motifs (CCHC) of the Hdh-vasa protein.

PCR amplification was used to isolate *vasa* and *PL10* fragments from embryonic *H. discus hannai* cDNA. Sequence comparisons with NCBI databases revealed that these fragments are orthologous to the *vasa* and *PL10* genes, respectively. Consequently, they were tentatively designated as *Hdh-vasa* and *Hdh-PL10*. The ORFs for Hdh-vasa and Hdh-PL10 encodes 853 aa and 749 aa, respectively; the ORF sequences can be found in GenBank under accession nos. PP891392 (*Hdh-vasa*) and PP891393 (*Hdh-PL10*). The predicted aa sequences of both Hdh-vasa and Hdh-PL10 shared all the nine motifs characteristic of the DEAD-box protein family (Q-motif, motif I, motif Ia, motif Ib, motif II, motif III, motif IV, motif V, and motif VI) (Figure 1 and Figure 2). The RG (arginine–glycine) and RGG (arginine-glycine-glycine) repeats at the N-terminal regions were both found in the Hdh-vasa and Hdh-PL10 (Figure 1 and Figure 2). An ARKF motif was also found in the vasa and PL10 (Figure 1 and Figure 2). The Hdh-vasa contains four tandem glycine-enriched sequences (Figure 1) and six tandem zinc-finger CCHC motifs (Figure 3) in its N-terminal region, which are absent in Hdh-PL10. Additionally, conserved Trp (W), Glu (E), and Asp (D) residues, characteristics of vasa family, are present in the C-terminal region of Hdh-vasa (Figure 1) but not in Hdh-PL10.

**Figure 4:**
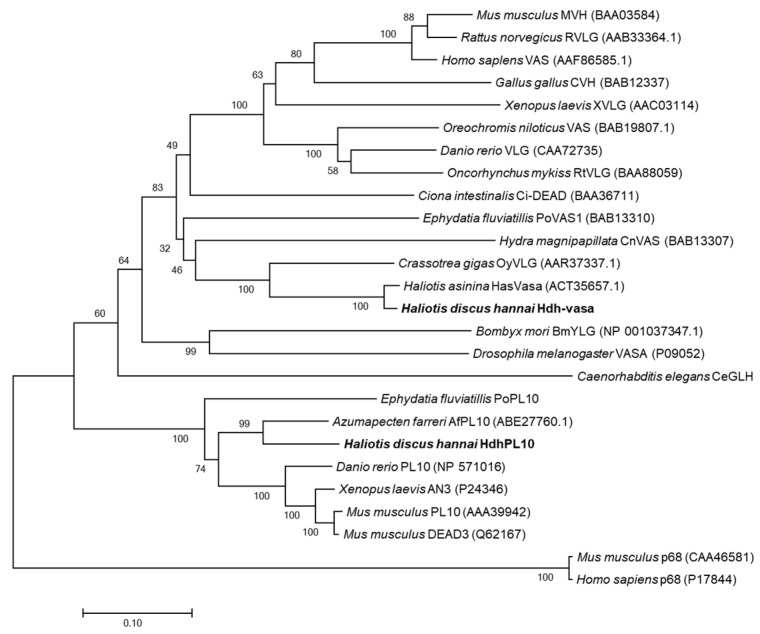
Phylogenetic analysis of vasa-related proteins. Each node displays the percentage values obtained from bootstrap analysis.

The *vasa* and *PL10* subfamilies belong to the same gene family, and the aa sequences of these two genes were compared in one evolutionary tree together (Figure 4). Phylogenetic analysis of the DEAD-box gene family identified the following three distinct clusters: the vasa, PL10, and P68 subfamilies (Figure 4). Hdh-vasa grouped with vasa proteins from other species, exhibiting robust bootstrap support, while Hdh-PL10 clustered with other PL10 proteins. In the vasa clade, Hdh-vasa clustered together with two other proteins sequences of mollusk species *Haliotis asinina* (HasVasa) and *Crassotrea gigas* (OyVLG) (Figure 4). Within the PL10 clade, Hdh-PL10 clusters with another mollusk protein sequence of *Azumapecten farreri* (AfPL10) (Figure 4). Therefore, phylogenetic analysis corroborates the initial orthology assignments, indicating that Hdh-vasa and Hdh-PL10 belong to the vasa and PL10 subfamilies, respectively.

### 3.2. Evaluation of Expression Stability of the Reference Genes for the Various Tissues and Different Embryonic Stages of H. discus hannai

#### 3.2.1. Selection of Reference Genes for Different Tissues

The expression levels of the ten candidate genes for different tissues were analyzed via RT-qPCR. The results of three statistical methods—GeNorm, NormFinder, and BestKeeper—are shown in Table 5. The GeNorm software assesses reference gene stability by calculating the average expression stability value (M) [37]. A lower M-value indicates greater expression stability. The software is set to a default critical value of 1.5. According to the analysis of GeNorm, the genes *ICG1* (M = 1.157), *ICG2* (M = 1.189), and *ICG7* (M = 1.193) showed good stability, while *β-actin* (M = 1.577) displayed unstable characteristics. The NormFinder software evaluates candidate reference genes according to their stability value, which takes into account both intra- and inter-group variations [38]. The gene exhibiting the lowest stability value demonstrates the greatest stability. According to the NormFinder analysis, *ICG1* (0.452) showed good stability. BestKeeper software assesses the stability of the reference gene’s expression using the correlation coefficient (r), standard deviation (SD), and coefficient of variation (CV) of the Cq values [39]. Higher r values and lower SD and CV values indicate a more stable expression. According to the analysis of the r values, *ICG2* (0.796), *ICG4* (0.755), and *ICG1* (0.740) showed good stability. According to the analysis of the SD values, *ICG1* (0.98), *ICG6* (1.02), and *ICG8* (1.06) showed good stability. According to the analysis of the CV values, *ICG1* (3.71), *ICG2* (4.19), and *ICG8* (4.21) showed good stability. Based on the overall data, our findings show that *ICG1*, which encodes the predicted protein KIAA1143 homolog (GenBank code: XM_067796144.1), is a highly stable and reliable reference gene.

#### 3.2.2. Selection of Candidate Reference Genes for Different Embryonic Developmental Stages

The expression levels of the ten candidate genes for different embryonic developmental stages were analyzed via RT-qPCR. The results of three statistical methods—GeNorm, NormFinder, and BestKeeper—are shown in Table 6. According to the analysis by GeNorm, *MZQ1* (M = 0.964), *MZQ4* (M = 1.023), and *MZQ2* (M = 1.037) showed good stability, while *MZQ5* (M = 1.505), *18s* rRNA (M = 1.881), and *β-actin* (M = 1.703) displayed unstable characteristic. According to the analysis by NormFinder, *MZQ1* (0.185), *MZQ4* (0.202), and *MZQ2* (0.258) showed good stability. According to the BestKeeper analysis of the r values, *MZQ1* (0.959), *MZQ4* (0.903), and *MZQ2* (0.873) showed good stability. According to the analysis of the SD values, *UBE2* (0.41), *MZQ2* (0.70), and *MZQ1* (0.72) showed good stability. According to the analysis of the CV values, *UBE2* (1.80), *MZQ2* (2.52), and *MZQ4* (2.90) showed good stability. Based on the overall data, our finding showed that *MZQ2*, which encodes GPI-anchored glycoprotein, was a highly stable and reliable reference gene.

### 3.3. Tissue-Specific Expression of Hdh-Vasa and Hdh-PL10

**Figure 5:**
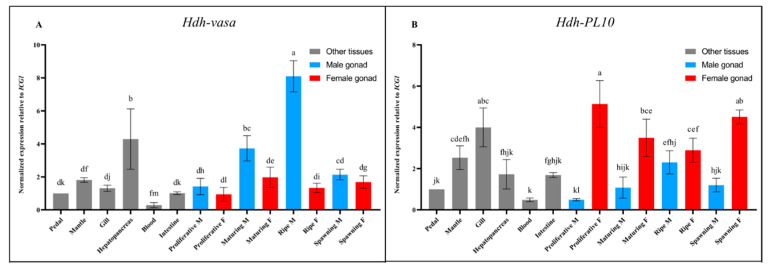
RT-qPCR results of various somatic tissues and gonads for (**A**) *Hdh-vasa* and (**B**) *Hdh-PL10*. The RT-qPCR values were determined relative to a reference sample, which included cDNA from all developmental stages involved in the experiment and were normalized against a reference gene index (*ICG1*). Significant differences are denoted by bars having distinct letters (*p* < 0.05). The error bars represent the standard error, which was calculated from three technical replicates.

To examine the tissue-specific expression of *Hdh-vasa* and *Hdh-PL10*, the pedal, mantle, gill, hepatopancreas, blood, intestine, and gonads were extracted from *H. discus hannai*. Interestingly, *Hdh-vasa* and *Hdh-PL10* were not only expressed in the gonads, but also in the somatic tissues (Figure 5). *Hdh-vasa* exhibited high expression in the testes and hepatopancreas while showing low expression in the ovaries, pedal, mantle, gill, and intestine. *Hdh-PL10* showed high expression in the ovaries, gill, mantle, and testes, while its expression was low in the pedal, hepatopancreas, and intestines (Figure 5). Interestingly, the expression of both *Hdh-vasa* and *Hdh-PL10* in the testes increased gradually during the spermatogenesis progress (from proliferative M to ripe M) and then decreased dramatically in spawning M phase. However, we did not observe this expression trend during oogenesis in both genes, although *Hdh-PL10* was expressed at a high level in the ovaries (Figure 5).

### 3.4. Spatiotemporal Expression of Hdh-Vasa and Hdh-PL10 Throughout Embryonic and Larval Development

**Figure 6:**
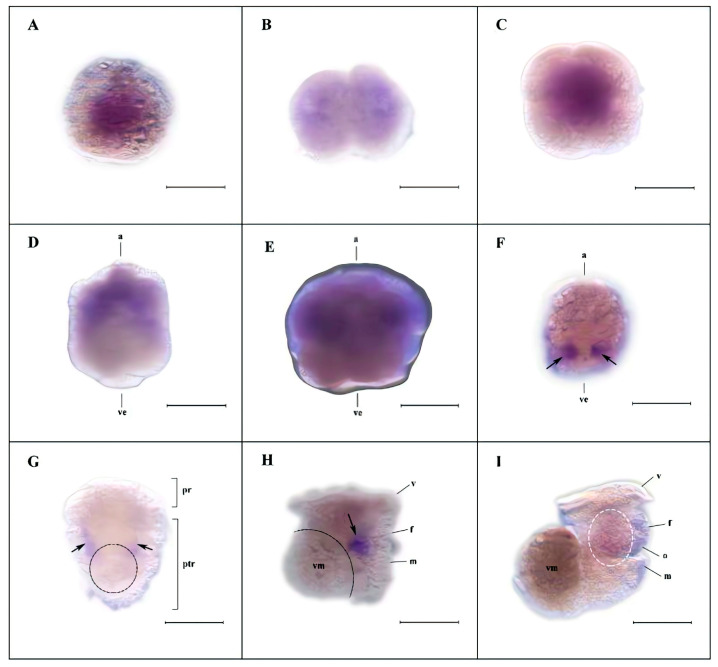
Expression of *Hdh-vasa* during embryonic and early larval development: (**A**–**I**) WMISH micrographs. (**A**–**C**) Lateral views—unfertilized egg (**A**), 2-cell embryo (**B**), and 4-cell embryo (**C**); (**D**,**E**) Lateral views, with the animal pole upwards and vegetal pole downwards; 8-cell embryo (**D**), 64-cell embryo (**E**); (**F**) Gastrulation stage, lateral view, with the animal pole upwards and vegetal pole downwards; black arrows indicate two positive signals; (**G**) trochophore stage, ventral view, with the pretrochal region upwards and the posttrochal region downwards; black dotted line indicates visceral mass, and black arrows indicate two positive signals; (**H**) Early veliger stage, right lateral view, the black dotted line indicates visceral mass, and black arrows indicate positive signals; (**I**) metaphase veliger stage, right lateral view, the white dotted line indicates positive signals. a, animal pole; ve, vegetal pole; pr, pretrochal region; ptr, posttrochal region; f, foot; vm, visceral mass; v, velum; m, mantle rudiment; and o, operculum. Scale bar: 200 μm.

**Figure 7:**
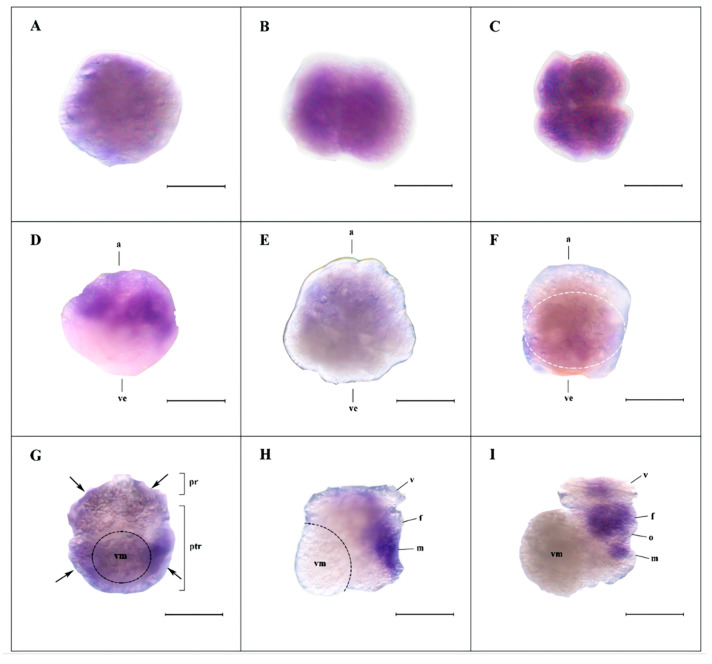
Expression of *Hdh-PL10* during embryonic and early larval development. (**A**–**I**) Representative WMISH micrographs. (**A**–**C**) lateral views—unfertilized egg (**A**), 2-cell embryo (**B**), 4-cell embryo (**C**); (**D**,**E**) lateral views, with the animal pole upwards and vegetal pole downwards—8-cell embryo (**D**) and 64-cell embryo (**E**); (**F**) gastrulation stage, lateral view, with the animal pole upwards and vegetal pole downwards; the white dotted line indicates positive signals; (**G**) trochophore stage, ventral view, with the pretrochal region upwards and posttrochal region downwards; black dotted line indicates visceral mass and black arrows indicate positive signals; (**H**) early veliger stage, right lateral view; the black dotted line indicates visceral mass; (**I**) metaphase veliger stage, right lateral view. a, Animal pole; ve, vegetal pole; pr, pretrochal region; ptr, posttrochal region; f, foot; vm, visceral mass; v, velum; m, mantle rudiment; o, operculum. Scale bar: 200 μm.

**Figure 8:**
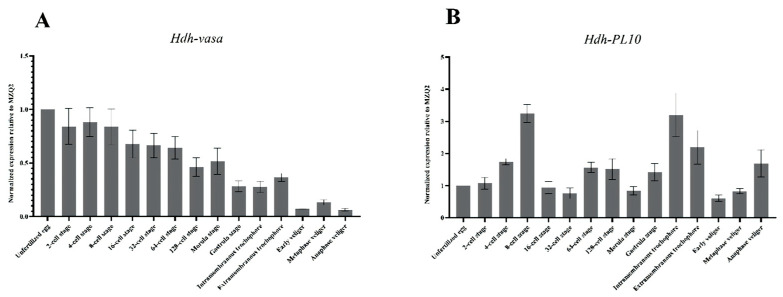
RT-qPCR results: (**A**) *Hdh-vasa* and (**B**) *Hdh-PL10*. Error bars represent the standard error estimated from three technical replicates.

The expression of *Hdh-vasa* and *Hdh-PL10* was analyzed during embryonic and larval development using WMISH (Figure 6 and Figure 7) and RT-qPCR (Figure 8). *Hdh-vasa* was initially observed in the unfertilized egg, primarily in the central region of the animal hemisphere and the animal portion of the vegetal hemisphere (Figure 6A). After the first cleavage, *Hdh-vasa* maintained the same localization pattern in each daughter cell, as observed in the unfertilized egg (Figure 6B). As cleavage progressed to the 4-cell stage, *Hdh-vasa* expression was present in all cells (Figure 6C). At the 8-cell stage, when four vegetal macromeres and four animal micromeres formed, *Hdh-vasa* transcripts were predominantly found in the micromeres (Figure 6D). This expression pattern persisted through the sixth cleavage, continuing into the 64-cell embryos (Figure 6E). Interestingly, at the gastrulation stage, the *Hdh-vasa* signals were found in the mesodermal region of the larva, showing strong expression in two bilaterally symmetric paraxial clusters (Figure 6F). After the formation and hatching of the trochophore larva, *Hdh-vasa* expression continued the same bilaterally symmetrical expressional pattern, localizing ventrally in the posttrochal mesodermal region (Figure 6G). As trochophore development to early veliger, *Hdh-vasa* expression was localized to the ventral foot and mantle region, near visceral mass region (Figure 6H). In metaphase veliger stage, *Hdh-vasa* expression spread to the entire foot and head regions, and the positive signal was weak (Figure 6I). The RT-qPCR during the embryonic and larval stages showed that *Hdh-vasa* was highly expressed in unfertilized oocytes. The level of its expression declined gradually throughout embryonic and larval development. During the veliger stages, the level of *Hdh-vasa* mRNA expression dropped significantly relative to earlier stages (Figure 8).

Current evidence suggests that *vasa*-like genes evolved from a subset of helicases within the *PL10* family [19]. Based on this, we opted to investigate the expression of *Hdh-PL10* to further explore the underlying relationship between *Hdh-vasa* and *Hdh-PL10*. From the unfertilized egg to the 64-cell stage (Figure 7A–E), the expression pattern of *Hdh-PL10* closely resembled that of *Hdh-vasa*. In the gastrulation stage, the *Hdh-PL10* signals were dispersed throughout the entire endodermal region (Figure 7F). In the trochophore stage, *Hdh-PL10* appeared to be expressed in ventrolateral ectoderm on either side of the presumptive midline, located in both pretrochal and posttrochal regions (Figure 7G). As trochophore development to early veliger, *Hdh-PL10* expression was confined to extensive areas surrounding the foot and mantle regions. (Figure 7H). This expression pattern was maintained to the metaphase veliger stage, with the *Hdh-PL10* transcripts being more concentrated in the foot region (Figure 7I). The expression levels of *Hdh-PL10* mRNA during the embryonic and larval stages, as determined by RT-qPCR, revealed that the *Hdh-PL10* transcripts were detectable from unfertilized egg through to the anaphase veliger stage, reaching a peak by the 8-cell and trochophore stages (Figure 8).

## 4. Discussion

The DEAD-box protein family contains nine highly conserved motifs, which are conserved across invertebrates and vertebrates [43]. Four of these motifs are recognized for their involvement the known functions of DEAD-box proteins, particularly in the activity of eIF-4A [44]. These proteins are believed to exhibit ATP-dependent RNA helicase activity, mediated by the ATP-A (AXXGXGKT) and ATP-B (DEAD) motifs, as well as to contribute to RNA unwinding, as indicated by the SAT and HRIGR motifs. The predicted aa sequences of Hdh-vasa and Hdh-PL10 contained these conserved domains, including both ATP-B (DEAD) and ATP-A (AQTGSGKT) motifs, along with the HRIGR and SAT motifs associated with RNA helicase activity. Multiple repeats of the RG and RGG motifs, which are putative RNA-binding motifs [45,46,47], were present in the N-terminal regions of both Hdh-vasa and Hdh-PL10. An ARKF motif, known as a characteristic feature of the vasa family [48], was also found in vasa and PL10. Therefore, these data strongly suggest that the characterized *Hdh-vasa* and *Hdh-PL10* sequences encode protein members of the DEAD-box family, exhibiting RNA-binding, ATPase, and helicase activities. Additionally, certain sequence features were present only in vasa and were not observed in PL10. A glycine (G)-rich region was present in the N-terminal site of Hdh-vasa, which is also found in a number of putative RNA-binding proteins [8]. In the C-terminal region of Hdh-vasa, six of the last eight aa were acidic (glutamate or aspartate residues), a feature commonly observed in various single-stranded nucleic-acid-binding proteins [46]. These discrepancies between Hdh-vasa and Hdh-PL10 may contribute to variations in their expression patterns and functional properties.

Vasa and PL10 are key components of an extensive regulatory network that plays a crucial role in the maintenance of germ stem cells, as well as in germline specification and differentiation. For this reason, it is expected that these two genes are predominantly expressed in germline cells, a phenomenon that has been extensively documented in previous studies [15,16,17,18,19,20,21,22]. For instance, in mollusks, as in other studied phyla, *vasa*-related genes are uniquely expressed in germline cells and are widely recognized as key germline markers [11,31,32,49,50]. Similarly, *PL10* is highly expressed in germline cells, from mammals such as mice to lower organisms like *Hydra* [19,51]. In our study, these two genes are expressed in both gonads (male and female) at all stages of adult development. The distinction is that *vasa* was highly expressed in the testes, whereas *PL10* exhibited high expression levels in both the ovaries and testes. Notably, the expression levels of *Hdh-vasa* and *Hdh-PL10* both increased gradually during male gonadal development (from proliferative M stage to ripe M stage), implying that both genes function synergistically in the development of spermatogenesis in *H. discus hannai*. Indeed, the role of these two genes and its homologues in spermatogenesis have been extensively reported in model animals [51,52,53,54].

However, it is important to note that the expression of *Hdh-vasa* was not restricted solely to the gonads but was also present in other tissues, including the hepatopancreas, pedal, mantle, gills, and intestines. The mRNA of *Hdh-PL10* was also not limited to the gonads but widely expressed in other tissues, including gill, mantle pedal, hepatopancreas, and intestine. The tissue expression pattern may reflect that the function of *vasa* and *PL10* are not limited to animal germlines but may also play diverse roles and participate in various biological processes in *H. discus hannai*. Studies in other animal species have shown that *vasa* and *PL10* genes play crucial roles in multipotent stem cells contributing to both somatic and germline lineages [55]. In *Hydra*, *vasa* and *PL10* are expressed in interstitial cells [19] and ectodermal epithelial cells [56,57], which generate germ cells and diverse somatic types. Similarly, in *Clytia hemisphaerica*, *vasa* and *PL10* are found in somatic stem cells located at the tentacle bulb base, from which tentacle nematocytes are derived [2]. In summary, our findings show that *vasa* and *PL10* are widely expressed in various tissues, suggesting they are linked to multipotent stem cells involved in essential biological processes beyond gametogenesis in *H. discus hannai*. Therefore, caution is recommended when utilizing them as adult germline markers for exploratory studies of this species.

Maternally inherited RNA and proteins control much of embryonic development, and these genes are referred to as maternal-effect genes. The effect of these mechanisms have been identified for a wide range of organisms [58,59,60,61,62]. Understanding the role of maternal effects is important for gaining insight into the basic mechanisms of inheritance. RT-qPCR analysis showed that *Hdh-vasa* expression peaked in unfertilized eggs and early development, then declined significantly in later stages. WMISH results also showed a large amount of *Hdh-vasa* observed in unfertilized oocytes and in first cleavage stage. Although *Hdh-PL10* exhibited a distinct developmental expression pattern, its abundant mRNA expression in unfertilized oocytes and at the first cleavage stage, is similar to *vasa*. Molluscan embryonic genome transcription initiates during the gastrula stage [63], with early developmental processes primarily relying on maternal genome-derived transcripts. Therefore, our results implied that *Hdh-vasa* and *Hdh-PL10* transcripts were maternally synthesized and, subsequently, transmitted to embryos to support early development. This suggests that *Hdh-vasa* and *Hdh-PL10* are potential maternal-effect genes in *H. discus hannai*. In *Drosophila*, *Danio rerio* and *Hydra*, *vasa*-related genes, have been demonstrated to be maternally inherited, playing a key role in germline differentiation during embryonic development prior to the onset of zygotic gene expression [19,46,64]. However, further mutation validation experiments are required to ascertain whether *Hdh-vasa* and *Hdh-PL10* function as maternal-effect genes.

PGCs are the earliest germ cells distinguishable from somatic cells during embryonic development. Germline development involves the specification and survival of PGCs during embryogenesis, as well as the sustained production of gametes throughout the adult reproductive lifespan. These processes are considered essential across all Metazoans [65]. PGC specification is typically categorized into the following two mechanisms: preformation (determinative mode), where PGCs are established early in development through maternally inherited factors, and epigenesis (inductive mode), where germ cells are not identifiable until later stages and arise through inductive signals from neighboring somatic cells [66]. Transcripts of many genes have been identified as germ plasm components, a granular cytoplasmic material that serves as a useful marker for identifying PGCs in many animal species [29]. Among these, *vasa* is a useful molecular marker and conserved across a wide range of animal classes [1]. Unlike traditional examples of preformation, where germ-cell-specific molecules (such as germ plasm components) are asymmetrically distributed to specific regions of the early embryo [67,68], *vasa* transcripts were located in all micromeres during the first six cleavages divisions in *H. discus hannai*. Instead, it was during late embryogenesis and the gastrulation stage that *Hdh-vasa* become restricted to specific cells. Given that *Hdh-vasa* is broadly expressed in the early embryo and symmetrically inherited during the initial cleavage stages, it seems improbable that the determination of PGCs in *H. discus hannai* is solely dependent on maternally inherited factors. Consequently, we propose that PGC establishment in *H. discus hannai* is more likely driven by inductive signals, following an epigenetic mode of regulation. We hypothesize the presence of undifferentiated multipotent cells expressing *Hdh-PL10*, which may play a broader role in stem cell function during early *H. discus hannai* embryogenesis. *Hdh-vasa*-expressing cells may constitute a subpopulation of these multipotent cells, which have the potential to give rise to both mesodermal tissue and PGCs. In this context, PGCs are determined from *vasa*-expressing cells at a certain stage of development. The co-expression of germ cell and stem cell markers is also observed in other organisms [19,24,69].

In the equally cleaving vetigastropod *H. asinina*, a similar pattern was observed, with *vasa* progressively enriching in the dorsal quadrant of the embryo, and PGC specification following an inductive signals (epigenesis) mechanism [50]. This “progressive restriction” could represent an ancestral mechanism of germ cell specification.

## 5. Conclusions

In conclusion, this study provides novel insights into the roles of *Hdh-vasa* and *Hdh-PL10*, members of the DEAD-box gene family, in germline development in *H. discus hannai*. Notably, the broad tissue expression patterns of these genes, beyond the gonads, suggest additional roles in multipotent stem cells and other biological processes. The maternal inheritance of Hdh-vasa and Hdh-PL10, alongside their significant expression in early developmental stages, supports their potential as maternal-effect genes that contribute to early embryonic development. Furthermore, the distinctive expression dynamics of *Hdh-vasa*, which is not asymmetrically inherited but rather gradually restricted during later development, suggest that PGC determination in *H. discus hannai* follows an epigenetic mode of regulation, driven by inductive signals.

In the future, further investigation into the interactions between *Hdh-vasa*, *Hdh-PL10*, and other key regulatory factors will shed light on their roles in embryonic development and germ cell formation. RNAi-mediated knockdown of *vasa* and *PL10* expression could help confirm their critical roles in germline cell specification and PGC formation. To gain a more comprehensive understanding of gene function, we could extend the sampling time to later developmental stages and apply advanced single-Cell transcriptomics to precisely capture gene expression dynamics with greater spatiotemporal resolution and reveal the regulation of *Hdh-vasa* and *Hdh-PL10* in PGC formation, differentiation, and migration across different developmental stages. These studies will enhance our understanding of the functional roles of these genes in germ cell development and contribute to advancing research on PGCs.

## Figures and Tables

**Table 1:** Primer design for ORF amplification of target genes.

Genes	Forward Primer (5′→3′)	Reverse Primer (5′→3′)	Amplicon Length (bp)
*vasa*	ATGAAAATGTGCCCTGTAAAGGCGCTGACCAATCAG	ACAAATTTCCCCTAACATTGATACCCAGGGGAACAGTG	2559
*PL10*	ATGAATCCCAATGATACCCATCAAAATGG	TTAGTTGCCCCACCAGTCGTTG	2247

**Table 2:** The sequences of the primers for the candidate genes of the various tissues.

Genes	GenBank Code	Forward Primer (5′→3′)	Reverse Primer (5′→3′)
*ICG1*	PV224500	CGACATCACAACAGAAGGCAAAA	TTTTCTTTCCTGATGTGCTCACC
*ICG2*	PV224501	CCAACAAGACCGTGATGAGAGA	GAGATCATACAGCTCTGCCCTC
*ICG3*	PV231213	AGGTAAGACTGGTGGGAAGGT	GCCATTTCTCACCAGCTTGTC
*ICG4*	PV224502	ACTCCAGCCCTTATCCTAGCA	CGCAACATTTGTCCGAGGATC
*ICG5*	PV224503	AGTATCAGACCAAGCTTGCAGAA	CGCCATCATTGTTCTCTTCTTCC
*ICG6*	PV224504	ATTGGTCCCCTCGGTTTGTC	GCCTGCCGATTCTGGATGAT
*ICG7*	PV224505	GCCAACACATTTGCAATCACC	TCCCTCTCCAGCAGCATTTG
*ICG8*	PV224506	TCCAGAGGACAAAGGAAGTGC	GACCTCCGACAAGACCTCTCT
*GAPDH*	PV224499	TGACAGTGGTGCCGAATATGT-	AACGTTCAGATCGGGTGTGTA
*β-actin*	AY380809	AACTTAGTCAGCGGCCG	AACCCGGCCTTACACAT

**Table 3:** The sequences of primers for the candidate genes in different embryonic development stages.

Genes	GenBank Code	Forward Primer (5′→3′)	Reverse Primer (5′→3′)
*MZQ1*	PV231213	TACTTTCTCTTCCTTGCCGACC	CCACCCTTTCTCCATTCTTCCA
*MZQ2*	PV224495	TTACCTGCCCTTGTCATGCA	AGCGGTTTCTTGTCACAGGT
*MZQ3*	PV224496	AGAAGTTGAAACAGTCGAAGCAGA	TGGTGAATTGTTCCAGATAAGCAG
*MZQ4*	PV224497	AAGCTTCGACGAAATGGTTGG	ATTCTTTCGCCATGTCAGGGT
*MZQ5*	PV224498	CGCCCATGAGAAGTGTGTTTT	TGCTCACTTTAGGCCTTCTGT
*UBE2*	KP698948	CCAAGCTCTTCTTAGTGCAC	CTCCCCACTTCCATCACTTT
*RPL8*	KP698947	TGGAAACTACGCCACAGTCA	GTCCTGCCTTCAACATTGGT
*GAPDH*	PV224499	TGACAGTGGTGCCGAATATGT-	AACGTTCAGATCGGGTGTGTA
*β-actin*	AY380809	AACTTAGTCAGCGGCCG	AACCCGGCCTTACACAT
*18S*	AY319437	CCGAGGGTCTCACTAAACCATTC′	ACTACAATGAAGACGAAGCCCA

**Table 4:** The sequences of the primers for the DIG-labeled RNA probes.

Genes	Forward Primer (5′→3′)	Reverse Primer (5′→3′)	Amplicon Length (bp)
*vasa*	CATTCTGGATGAGGCTGACAGGATG	GGCGGCAACTGATGTGGCAATA	472
*PL10*	AGGACTACTTTGGGTTGTGTCAGGA	CCGATGGCTTGGCAGATTGGAC	529

**Table 5:** Candidate reference genes for the various tissues by GeNorm, NormFinder, and BestKeeper.

Gene	GeNorm	NormFinder	BestKeeper		
	(M Value)	(Stability Value)	(r)	(SD)	(CV)
*ICG1*	1.157	0.452	0.740	0.98	3.71
*ICG2*	1.189	0.504	0.796	1.11	4.19
*ICG3*	1.403	0.538	0.616	1.52	6.56
*ICG4*	1.340	0.549	0.755	1.28	4.52
*ICG5*	1.236	0.575	0.619	1.08	4.41
*ICG6*	1.203	0.656	0.681	1.02	4.86
*ICG7*	1.193	0.690	0.707	1.07	4.97
*ICG8*	1.306	0.714	0.555	1.06	4.21
*β-actin*	1.577	0.778	0.234	1.12	5.36
*GAPDH*	1.358	0.917	0.612	1.20	5.38

**Table 6:** Results of the candidate reference genes for various embryonic stages by GeNorm, NormFinder, and BestKeeper.

Gene	GeNorm	NormFinder	BestKeeper		
	(M Value)	(Stability Value)	(r)	(SD)	(CV)
*MZQ1*	0.964	0.185	0.959	0.72	3.66
*MZQ2*	1.037	0.202	0.873	0.70	2.52
*MZQ3*	1.247	0.258	0.794	0.95	3.50
*MZQ4*	1.023	0.443	0.903	0.76	2.90
*MZQ5*	1.505	0.562	0.537	1.24	4.63
*RPL8*	1.227	0.583	0.672	0.84	3.79
*UBE2*	1.131	0.697	0.618	0.41	1.80
*18s rRNA*	1.881	0.881	0.592	1.43	7.42
*β-actin*	1.703	1.024	0.726	1.50	6.81
*GAPDH*	1.313	1.208	0.505	0.86	3.52

## Data Availability

All relevant data are available from the corresponding author upon reasonable request.
